# Clozapine prescription rates in Southeast Europe: A cross-sectional study

**DOI:** 10.3389/fpsyt.2023.1123246

**Published:** 2023-04-11

**Authors:** Manuela Russo, Dragana Ignjatovic-Ristic, Dan Cohen, Aliriza Arenliu, Stojan Bajraktarov, Alma Dzubur Kulenovic, Lidija Injac Stevovic, Nadja Maric, Antoni Novotni, Nikolina Jovanovic

**Affiliations:** ^1^Unit for Social and Community Psychiatry, Wolfson Institute of Population Health, Queen Mary University of London, London, United Kingdom; ^2^Centre for Implementation Science, Health Services and Population Research Department, Institute of Psychiatry, Psychology & Neuroscience, King’s College London, London, United Kingdom; ^3^Department of Psychiatry, Faculty of Medical Sciences, University of Kragujevac, Kragujevac, Serbia; ^4^MHO North-Holland North, Heerhugowaard, Netherlands; ^5^Department of Psychology, University of Pristina, Pristina, Kosovo by United Nations Resolution; ^6^University Clinic of Psychiatry, Skopje, North Macedonia; ^7^Clinical Center University of Sarajevo, Sarajevo, Bosnia and Herzegovina; ^8^Psychiatric Clinic, University of Montenegro, Podgorica, Montenegro; ^9^Faculty of Medicine, Institute of Mental Health, University of Belgrade, Belgrade, Serbia

**Keywords:** clozapine, clinical recommendations, pattern of use, psychosis, low- and middle-income countries

## Abstract

**Introduction:**

International reports indicate that clozapine is under prescribed. Yet, this has not been explored in Southeast European (SEE) countries. This cross-sectional study investigates clozapine prescription rates in a sample of 401 outpatients with psychosis from Bosnia and Herzegovina, Kosovo by United Nations resolution, North Macedonia, Montenegro and Serbia.

**Methods:**

Descriptive analysis was used to explore clozapine prescription rates; daily antipsychotic dosage was calculated and converted into olanzapine equivalents. Patients receiving clozapine were compared to those not receiving clozapine; next those that were on clozapine monotherapy were compared to those who were on clozapine polytherapy regime.

**Results:**

It was showed that clozapine was prescribed to 37.7% of patients (with cross-country variation: from 25% in North Macedonia to 43.8% in Montenegro), with average dose of 130.7 mg/daily. The majority of patients on clozapine (70.5%) were prescribed at least one more antipsychotic (the most frequent combination was with haloperidol).

**Discussion:**

Our findings suggested that clozapine prescription rate in SEE outpatients is higher than in Western Europe. The average dose is significantly below the optimal therapeutic dosage recommended by clinical guidelines, and clozapine polytherapy is common. This might indicate that clozapine is prescribed mainly for its sedative effect rather than antipsychotic. We hope that this finding will be taken up by relevant stakeholders to address this non-evidence-based practice.

## 1. Introduction

Clozapine is a second-generation antipsychotic (SGA) drug recommended for treatment-resistant schizophrenia and the best among oral antipsychotic (AP) medications to improve positive and negative symptoms of schizophrenia (1, 2), prevent suicide, and reduce aggressive behavior (3, 4). Meta-analytic data showed that clozapine has the highest antipsychotic effect among 32 antipsychotics, the lowest incidence of akathisia (1) and rehospitalization, and reduced mortality (5–7). Beyond clinical benefits, there is also evidence suggesting that clozapine could be very cost-effective for the management of schizophrenia (8, 9).

Despite clinical and economic advantages, prescription rates for clozapine are low and there is a diffuse non-adherence to standard clinical guidelines. Clozapine’s potentially serious and life-threatening adverse events (i.e., agranulocytosis, neutropenia, and cardiac and metabolic complications) (10), along with inadequate knowledge and experience with the drug, patient’s noncompliance, and a lack of resources to blood monitoring and treatment protocol (11–14) can explain a diffuse prescription reluctancy among clinicians (3, 15–17) despite there is evidence that monitoring guidelines can be effective in the timely detection of treatment emergent neutropenia or agranulocytosis (18, 19). Nonadherence to clinical guidelines in clozapine prescription is a common practice across countries, regardless of their economies and healthcare systems. Data showed wide variation in prescription rates, from relatively expected prevalence rates of 0.1–0.2% for every 100 people in Finland and New Zealand (which is aligned with the fact that schizophrenia has a prevalence of approximately 1% and that about 30% of that is composed of treatment-resistant Schizophrenia (TRS)) to lower or null in some other countries (i.e., 0.03–0.06% in Spain and Japan, respectively) (3, 20). Similarly, data from 14 Asian countries (3,774 patients) found not only large variations in prescription (from 3% in Japan to 32% in Hong Kong) but also in the mean daily dosage, which was 198 mg (±167) (from 58 mg in Indonesia to 423 mg in Korea) (17). Variations within the same country are also often reported in Denmark, Norway, Spain, England, Wales, and the United States (16, 20–24).

While most of the available data derives from high-income countries (HICs), data about clozapine prescription in low- and middle-income countries (LMICs) is not systematically collected and is overall scanty. Research from Indonesia showed that clozapine was the second most commonly prescribed SGA in 38% of patients with schizophrenia, mainly prescribed with risperidone (25), while in India, it was prescribed only in 11.2% of patients (12). Unexpectedly, data from Uzbekistan showed that clozapine was the most commonly prescribed AP in up to 66% of patients with schizophrenia (26) but at a low dosage of 69 mg/day. Data from Central and Eastern Europe examining 961 medical records from inpatients and outpatients with schizophrenia (less than 20% with up to 6 months from first diagnosis) showed that prescription rates in TRS varied from 11% in Hungary, to up to 35% in Poland, Estonia, and Slovakia, 41% in Croatia, and above 60% in Slovenia and Serbia (27). With the exception of Serbia and Montenegro, suggesting an increase in clozapine prescription over the period 2000–2005 (15), there is a lack of data on clozapine prescription in Southeast European (SEE) countries. In Serbia, clozapine was found to be the second most prescribed AP as a second-line treatment for schizophrenia (35%) (27) and the main drug used (73%) in treatment-resistant schizophrenia. A recent survey about the prescription attitude toward clozapine in Serbia found that 68% of psychiatrists indicated fear of agranulocytosis as the greatest barrier to prescription, followed by weight gain (56%) and sedation (39%) (28). Moreover, other data from Serbia suggested that only 4% of patients reported severe side effects and 32% experienced moderate side effects. It was also found that there was only a weak correlation between side effects and clozapine dosage (*r* = 0.2) and that, due to the regular and systematic monitoring of side effects, patients reported an improved relationship with their treating clinicians (29).

In summary, although nonadherence to clinical guidelines seems to be an ubiquitous challenge regardless of countries’ economies, very little is known about clozapine prescription in LMICs from SEE. As the region is considered a blind spot in mental healthcare practice and research (30), it would be critical to have a better understanding of clozapine prescription in the area.

The aim of this study was to explore clozapine prescriptions across five LMICs in SEE (Bosnia and Herzegovina, Kosovo by United Nations resolution, Montenegro, North Macedonia, and Serbia) using a cross-sectional sample of 401 participants with schizophrenia and schizoaffective disorders from outpatient services. Specifically, we examined the proportion of patients who were prescribed clozapine (monotherapy and polytherapy with other APs) and concomitant psychotropic medications. Finally, we conducted an exploratory analysis on clinical symptoms and subjective satisfaction with services and quality of life between participants taking clozapine and those not taking clozapine across the five countries.

## 2. Methods

### 2.1. Sample

Data was gathered, as a part of secondary analysis, on 401 research participants with a diagnosis of schizophrenia and schizoaffective disorder (ICD-10 F20-25) across five SEE countries as part of a large multisite study (31). Eligibility criteria for taking part in the original study were a primary diagnosis of a psychotic disorder (ICD-10 codes F20–25), age 18–65, history of at least one psychiatric hospital admission in their lifetime, and capacity to provide informed consent. Exclusion criteria were a diagnosis of organic brain disorders, severe cognitive deficits, and the inability to provide informed consent.

### 2.2. Procedures

Information about psychotropic medications prescribed up to 6 months prior to the interview was obtained during a face-to-face interview, which was then cross-checked with medical records. All psychotropic medications’ dosages were reported in mg per day.

Sociodemographic information was collected as part of a comprehensive assessment. Data on clinical symptoms were collected using the researcher-administered 24-item Brief Psychiatric Rating Scale (BPRS; 32), from which a total score and subscale scores (anxiety/depression, positive symptoms, negative symptoms, hostility, and activation) were obtained; and the Clinical Assessment Interview for Negative Symptoms (CAINS; 33) from which the Motivation and Pleasure (MAP) and the Expression (EXP) subscales were calculated. For both the BPRS and CAINS, a higher score indicates more severe symptoms. The Manchester Short Assessment of Quality of Life (MANSA; 34) was used to measure subjective satisfaction with the quality of life and the Client Satisfaction Questionnaire (CSQ-8) measured satisfaction with services. On both of these latter measures, higher scores indicated higher satisfaction.

### 2.3. Data analysis

A descriptive analysis of sociodemographic, clinical characteristics, and clozapine prescription (used as a dichotomous variable: Y/N) for the whole sample was done by reporting frequency (absolute value and percentage), mean, and standard deviation (SD) as appropriate. Daily AP dosage was calculated and converted into olanzapine (OLA) equivalents (35, 36). When more than one AP was prescribed, OLA equivalent dosages for each AP were summed. Following the Canadian Psychiatric Association [CPA; (10)], a chlorpromazine equivalent dose of 400 mg/day, corresponding to 13.2 mg OLA equivalents, was considered a high maintenance dose. Comparisons were done across the five countries using analysis of variance and Chi-square (or Fisher’s exact test when expected frequencies were less than 5) for continuous and categorical variables, respectively. Next, analyses focused on the subsamples of participants who were prescribed clozapine (CLZ-Y) by country: comparisons were done in terms of clozapine’s daily dosage, range of daily dosage, proportion of participants on clozapine monotherapy (CLZ-M) and clozapine polytherapy (CLZ-P), and association with other psychotropic medications other than Aps, which were anxiolytics and antidepressants. The two groups (CLZ-M and CLZ-P) were then compared at clinical levels (as measured by the BPRS and CAINS), subjective quality of life (MANSA), and satisfaction with services (CSQ-8). Finally, for each country, it was explored whether clinical symptoms (as measured by the BPRS and CAINS) and satisfaction with life and health services (MANSA and CSQ-8, respectively) were (1) different between participants on clozapine (CLZ-Y) and those not on clozapine (CLZ-N) using an independent sample t-test; and (2) associated with dosages of clozapine using Spearman correlation. All significant differences emerged from analysis of variance were followed-up with *post-hoc* test using Tukey’s Honest Significant Difference at *p* < 0.05. SPSS version 27 was used to conduct analyses.

## 3. Results

### 3.1. Whole sample

The sample included a total of 401 research participants (62 from Bosnia and Herzegovina, 102 from Kosovo by United Nations resolution, 105 from Montenegro, 64 from North Macedonia, and 68 from Serbia; Table 1). Women were 163 (40.6%) of the whole sample. The mean age at the time of diagnosis was 42.0 years (SD = 11.0); 370 (92.3%) of the subjects had a diagnosis of schizophrenia, and 31 (7.7%) had schizoaffective disorder. Participants from Montenegro reported an older age at the time of their first diagnosis of a psychotic disorder compared to other countries (*p* < 0.001). A statistically significant difference was also found in the number of lifetime psychiatric hospitalizations (*p* < 0.001), which was higher in Bosnia and Herzegovina, and Serbia compared to other countries (Table 1). The OLA equivalent dosage in the whole sample was 12.3 mg/day; specifically, it was found that OLA equivalent dose in Kosovo by United Nations resolution (8.6 mg/day) was lower than in Montenegro, North Macedonia, and Serbia. Clozapine was prescribed in 149 subjects (37.2%) of the whole sample with no difference in prescription rates across countries (Table 1). When comparing the two groups of patients, with clozapine and without clozapine treatment, we found no statistically significant differences in demographic characteristics (i.e., age and gender) or clinical characteristics (diagnosis of affective and non-affective psychosis, age at the time of diagnosis, or OLA equivalent dosage). However, the number of psychiatric hospitalizations was significantly higher in patients on clozapine [4.6 ± 3.8 vs. 0.2.8 ± 2.8; *t*(326) = −5.0; *p* < 0.001]. Severe negative symptoms, derived from the motivation and pleasure (MAP) subscale of the CAINS, were increased nonsignificantly [17.7 ± 8.0 vs. 16.2 ± 8.6; *t*(384) = −1.75; *p* = 0.080].

**Table 1:** Description of sociodemographic and clinical characteristics in the whole sample (first column) and comparison across the five countries.

	Whole sample	Bosnia and Herzegovina^a^	Kosovo by United Nations resolution^b^	Montenegro^c^	North Macedonia^d^	Serbia^e^	Statistics
	(*n* = 401)	(*n* = 62)	(*n* = 102)	(*n* = 105)	(*n* = 64)	(*n* = 68)	
Age, mean (SD)	42.0 (11.0)	39.4 (12.2)	43.5 (9.7)	41.5 (11.9)	42.2 (10.1)	43.0 (11.0)	*F*(4) = 1.52; *p* = 0.196
Gender, n (%)							
Women	163 (40.6)	31 (50.0)	32 (31.4)	50 (47.6)	23 (35.9)	27 (39.7)	Chi^2^(4) = 8.6; *p* = 0.072
Men	238 (59.4)	31 (50.0)	70 (68.6)	55 (52.4)	41 (64.1)	41 (60.3)	
Diagnosis, n (%)							
Non-affective psychosis	370 (92.3)	58 (93.5)	102 (100.0)	92 (87.6)	61 (95.3)	57 (83.8)	Fisher Exact test = 22.3; *p* < 0.001
Schizoaffective	31 (7.7)	4 (6.5)	0 (0.0)	13 (12.4)	3 (4.7)	11 (16.2)	
Age at first diagnosis, mean (SD)	30.6 (10.4)	27.2 (11.2)	29.9 (9.7)	35.8 (12.2)	29.6 (7.1)	29.5 (8.9)	*F*(4) = 7.10; *p* < 0.001
							c > a = b = d = e
Lifetime psychiatric hospital admissions, mean (SD)	3.5 (3.3)	4.2 (4.3)	2.1 (2.8)	3.2 (3.0)	2.6 (2.4)	4.7 (3.54)	*F*(4) = 5.8; *p* < 0.001
							a > b; e > b = c = d
Olanzapine equivalents, mg/day dose, mean (SD)	12.3 (8.5)	11.7 (7.2)	8.6 (6.6)	12.7 (9.8)	13.5 (7.4)	15.6 (8.7)	*F*(4) = 7.6; *p* < 0.001
							b < c = d = e
Clozapine prescription, n (%)							
Yes	149 (37.2)	24 (38.7)	37 (36.3)	46 (43.8)	16 (25.0)	26 (38.2)	Chi^2^(4) = 6.2; *p* = 0.187
No	252 (62.8)	38 (61.3)	65 (63.7)	59 (56.2)	48 (75.0)	42 (61.8)	

### 3.2. Subsample of participants on clozapine

The average daily dose of clozapine was 130.7 mg (SD = 103.9 mg, ranging from 5 mg to 600 mg/day). The highest dose was found in the Serbian sample (220.4 mg, SD = 145.3 mg), which was a statistically significant difference from other countries (*p* < 0.001). Similarly, the dosage ranges were statistically different across countries, with Serbia reporting the highest proportion of participants (34.6%) with at least 251 mg/day, while for all other countries, the majority of participants had prescriptions lower than 100 mg/day. Of the 149 participants on clozapine, 44 (29.5%) were on clozapine monotherapy, and 105 (70.5%) were on clozapine polytherapy. The two groups showed statistically significant differences in OLA equivalents that were higher in the CLZ polytherapy group compared to the CLZ monotherapy group (15.3 ± 9.6 vs. 5.9 ± 4.8, respectively, *t*(143) = −6.2, *p* < 0.001). In the CLZ polytherapy group, the most frequent clozapine combination was with haloperidol (*n* = 49, 46.7%), followed by fluphenazine (*n* = 23, 21.9%) (Table 2). These combinations were reported across all countries except for Serbia, where the combination of clozapine and fluphenazine (38.9%) was the most commonly used. In the group of participants who were prescribed clozapine, 86 (57.7%) were also prescribed anxiolytics, and 24 (16.1%) were prescribed antidepressants. Differences across countries emerged in the prescription of anxiolytics (*p* < 0.001): specifically, while only 8.3% of participants were prescribed anxiolytics in Bosnia and Herzegovina, the proportion was much higher (from 43.2 to 80.4%) in all the other countries (Table 2).

**Table 2:** Description of rates of clozapine and other psychotropic drugs other than antipsychotics in the subsample of participants prescribed with clozapine and comparison across the five countries.

	Subsample on clozapine	Bosnia and Herzegovina	Kosovo by United Nations resolution	Montenegro	North Macedonia	Serbia	Statistics
Clozapine, mg/day dose, mean (SD)	*N* = 144	*N* = 24	*N* = 34	*N* = 46	*N* = 15	*N* = 26	
							*F*(4) = 8.4; *p* < 0.001
	130.7 (103.9)	114.8 (65.5)	92.6 (51.3)	123.3 (94.0)	101.7 (97.5)	225.0 (146.1)	e > a = b = c = d
Clozapine dose’s range, n (%)	*N* = 144	*N* = 24	*N* = 34	*N* = 45	*N* = 15	*N* = 26	Chi^2^(16) = 46.5; *p* < 0.001
Up to 50 mg/day	42 (29.2)	4 (16.7)	11 (32.4)	18 (40.0)	7 (46.7)	2 (7.7)	
51 mg – 100 mg/day	48 (33.3)	12 (50.0)	18 (52.9)	8 (17.8)	4 (26.7)	6 (23.1)	
101 mg – 200 mg/day	32 (22.2)	6 (25.0)	5 (14.7)	13 (28.9)	2 (13.3)	6 (23.1)	
201 mg-250 mg/day	8 (5.6)	1 (4.2)	0 (0.0)	3 (6.7)	1 (6.7)	3 (11.5)	
251 mg/day and above	14 (9.7)	1 (4.2)	0 (0.0)	3 (2.1)	1 (6.7)	9 (34.6)	
Clozapine regime, n (%)	*N* = 149	*N* = 24	*N* = 37	*N* = 46	*N* = 16	*N* = 26	Chi^2^(4) = 3.0; *p* = 0.557
CLZ Monotherapy	44 (29.5)	8 (33.3)	14 (37.8)	11 (23.9)	3 (18.8)	8 (30.8)	
CLZ Polytherapy	105 (70.5)	16 (66.7)	23 (62.2)	35 (76.1)	13 (81.3)	18 (69.2)	
Olanzapine Equivalents, mg/day dose, mean (SD)	*N* = 145	*N* = 24	*N* = 35	*N* = 45	*N* = 15	*N* = 26	*F*(9) = 7.1; *p* = 0.021
CLZ Monotherapy	5.9 (4.8)	4.1 (2.1)	4.7 (2.7)	4.6 (4.4)	10.4 (7.5)	9.5 (6.6)	
CLZ Polytherapy	15.3 (9.6)	12.4 (7.0)	10.9 (9.6)	17.8 (10.8)	12.9 (7.0)	20.0 (8.3)	
Clozapine polytherapy, n (%)	*N* = 105	*N* = 16	*N* = 23	*N* = 35	*N* = 13	*N* = 18	Chi^2^(16) = 35.9; *p* = 0.003
Clozapine + Haloperidol	49 (46.7)	11 (68.8)	7 (30.4)	18 (51.4)	9 (69.2)	4 (22.2)	
Clozapine + Fluphenazine	23 (21.9)	0 (0.0)	6 (26.1)	8 (22.9)	2 (15.4)	7 (38.9)	
Clozapine + Risperidone	16 (15.2)	2 (12.5)	3 (13.0)	5 (14.3)	2 (15.4)	4 (22.2)	
Clozapine + Aripiprazole	5 (4.8)	2 (12.5)	0 (0.0)	0 (0.0)	0 (0.0)	3 (16.7)	
Clozapine + any other AP	12 (11.4)	1 (6.3)	7 (30.4)	4 (11.4)	0 (0.0)	0 (0.0)	
Prescribed anxiolytics, n (%)	*N* = 149	*N* = 24	*N* = 37	*N* = 46	*N* = 16	*N* = 26	Fisher Exact test = 43.0; *p* < 0.001
Yes	86 (57.7)	2 (8.3)	16 (43.2)	37 (80.4)	12 (75.0)	19 (73.1)	
No	63 (42.3)	22 (91.7)	21 (56.8)	9 (19.6)	4 (25.0)	7 (26.9)	
Prescribed antidepressants, n (%)	*N* = 149	*N* = 24	*N* = 37	*N* = 46	*N* = 16	*N* = 26	Fisher Exact test = 7.4; *p* = 0.105
Yes	24 (16.1)	2 (8.3)	6 (16.2)	13 (28.3)	1 (93.8)	2 (7.7)	
No	125 (83.9)	22 (91.7)	31 (83.8)	33 (71.7)	15 (6.3)	24 (92.3)	

### 3.3. Association between clozapine and clinical characteristics

It was found that the CLZ-M and the CLZ-P did not differ in clinical symptoms’ severity and subjective quality of life, but only in satisfaction with services (CSQ-8), with the CLZ-M group being less satisfied than the CLZ-P group [27.3 ± 3.9 vs. 25.1 ± 5.6, *t*(147) = 2.3, *p* = 0.021]. Comparisons between CLZ-Y and CLZ-N across countries showed that people taking clozapine reported lower satisfaction with services in Montenegro (*p* = 0.027) and Serbia (*p* = 0.047) and more severe negative symptoms (MAP subscale of CAINS) in North Macedonia (*p* = 0.044) and Serbia (*p* = 0.042) compared to the CLZ-N group (Table 3). A negative association was found between a higher dose of clozapine and both subjective quality of life (*r* = −0.654, *p* = 0.008) in North Macedonia and more severe negative symptoms (MAP subscale; *r* = −0.372, *p* = 0.039) in Kosovo by United Nations resolution.

**Table 3:** Comparison between participants on clozapine (CLZ-Y) and not on clozapine (CLZ-N) across clinical symptoms and satisfaction with services.

	Bosnia and Herzegovina		Kosovo by United Nations resolution		Montenegro		North Macedonia		Serbia	
	CLZ-Y (*n* = 24)	CLZ-N (*n* = 38)	CLZ-Y (*n* = 37)	CLZ-N (*n* = 65)	CLZ-Y (*n* = 46)	CLZ-N (*n* = 59)	CLZ-Y (*n* = 16)	CLZ-N (*n* = 48)	CLZ-Y (*n* = 26)	CLZ-N (*n* = 42)
BPRS, mean (SD)										
Anxiety and Depression	7.4 (3.9)	7.5 (3.0)	11.9 (5.4)	12.3 (4.8)	8.4 (4.3)	8.6 (3.8)	9.9 (4.3)	10.2 (4.4)	11.2 (4.3)	10.5 (4.4)
Positive symptoms	5.5 (2.5)	5.6 (3.3)	6.1 (2.5)	7.2 (3.5)	6.8 (3.7)	6.1 (3.2)	5.9 (2.6)	6.7 (3.7)	6.5 (3.5)	5.7 (2.2)
Negative symptoms	5.5 (2.8)	5.4 (2.3)	7.9 (3.4)	7.7 (3.7)	5.8 (3.2)	6.7 (3.9)	7.3 (3.5)	7.0 (3.6)	7.3 (3.1)	6.9 (3.5)
Hostility	4.2 (2.0)	4.3 (2.5)	5.3 (2.3)	5.9 (2.7)	4.2 (1.0)	4.7 (2.0)	5.2 (2.1)	4.8 (1.7)	5.3 (1.9)	5.2 (2.3)
Activation	4.8 (1.6)	4.4 (1.8)	5.6 (2.5)	5.4 (2.1)	3.8 (1.2)	4.4 (2.3)	5.8 (1.9)	5.2 (1.8)	5.2 (2.0)	5.0 (1.7)
Total score	36.4 (13.0)	36.4 (12.0)	49.3 (11.8)	51.7 (13.7)	38.5 (8.1)	40.4 (10.0)	45.4 (9.3)	44.0 (12.4)	46.1 (10.7)	43.7 (11.0)
CAINS, mean (SD)	9.0 (9.4)	8.8 (8.7)	16.8 (5.5)	17.3 (7.9)	20.7 (7.1)	20.3 (7.2)	18.6 (7.1)	14.3 (7.3)	21.0 (5.6)	17.6 (7.8)
Motivation and Pleasure										
							*t* = (58) = −2.06; *p* = 0.044		*t* = (65.6) = −2.08; *p* = 0.042	
Expression	3.6 (3.7)	3.6 (3.8)	5.0 (3.4)	5.2 (3.8)	4.2 (4.1)	4.8 (4.7)	7.1 (4.6)	5.1 (4.4)	5.4 (3.4)	4.8 (3.4)
MANSA tot, mean (SD)	4.9 (0.9)	5.0 (0.9)	4.1 (0.9)	4.1 (1.0)	4.4 (1.0)	4.4 (1.1)	4.6 (0.6)	4.7 (0.8)	4.3 (0.7)	4.3 (0.7)
CSQ-8, mean (SD)	26.7 (7.1)	28.6 (4.9)	26.2 (5.0)	27.8 (3.2)	25.2 (5.9)	27.5 (4.8)	26.0 (3.0)	27.6 (4.1)	25.2 (2.9)	26.9 (3.5)
					*t*(103) = 2.25; *p* = 0.027				*t*(66) = 2.02; *p* = 0.047	

Only comparisons that resulted as statistically significant at *p* < 0.05 are reported in the table.

## 4. Discussion

Results from this multisite, cross-sectional study, which included 401 research participants with schizophrenia and schizoaffective disorder from outpatient mental health services in five SEE countries, suggested that clozapine is subjected to a wide variation in prescription and low adherence to clinical guidelines. Results showed that clozapine was prescribed in almost 38% of the whole sample. The prescription of clozapine varied across countries, ranging from 25% in North Macedonia to 36.3% in Kosovo by United Nations resolution, 38.2% in Serbia, 38.7% in Bosnia and Herzegovina, and 43.8% in Montenegro. Data from North Macedonia reflect what emerged from a previous study by Szkultecka-Debek et al. (27), showing that clozapine was prescribed in about 24% of patients across seven Central and Eastern European countries, while prescription rates were higher across all other countries in our study. These data are unexpected compared to a general international trend to underprescribe clozapine (3, 17, 22). Despite the fact that the percentage of patients (37.7%) on clozapine in our sample is slightly above but nevertheless closer to the expected proportion of one-third of total patients with schizophrenia that are usually TRS (37), we cannot assume that patients in our sample belong to the TRS group. Indeed, only in about less than 10% of them was clozapine prescribed with a daily dosage of at least 250 mg, while most patients had a daily dosage of 130.7 mg, which is lower than what clinical guidelines indicate as the optimal (therapeutic) dosage of 300 mg/day for clozapine in adults aged 18–59 years (38, 39). Furthermore, considerable variations emerged across countries, from 92.6 mg (Kosovo by United Nations resolution) to 220.4 mg (Serbia). Although there is evidence that genetic (including ethnicity), personal, and clinical variables (i.e., sex, smoking status, and obesity) influence the serum level of clozapine so that a lower dosage based on specific subjects’ characteristics is advisable (40–42), there is no ground to support such an option in our sample. Therefore, we could assume that the lower dosages of clozapine found in our study might indicate an off-label prescription of the drug to possibly exert a sedative effect for managing non-psychotic symptoms for which clozapine is shown to be effective, such as hostility, verbal and physical aggression, suicidality, and possibly movement disorders as side effects of other antipsychotic medications (43–46). Moreover, results about the association of clozapine prescription with clinical symptoms also suggested that it was not associated with any symptomatic advantage. Conversely, in North Macedonia and Serbia, the group on clozapine reported higher negative symptoms, while lower satisfaction with services was reported in patients from Montenegro and Serbia.

Our data also showed that almost ¾ of the participants who were prescribed clozapine were on a polytherapy regime (70.5%; *n* = 105), with another AP medication: the most frequent combination was between clozapine and haloperidol (46.7%), followed by clozapine and fluphenazine (21.9%). Levels of olanzapine equivalents were higher in the group of individuals who were on clozapine polytherapy compared to those on clozapine monotherapy. Specifically, olanzapine equivalents in the clozapine polytherapy group were 15.3 mg/day vs. 5.9 mg/day in the clozapine monotherapy group, which is higher than the 13.2 mg/day that is considered a high maintenance dose for clozapine (10). Even higher olanzapine equivalent dosages were found in the clozapine polytherapy group in Montenegro and Serbia (17.8 and 20 mg/day, respectively). This result is in line with trends showing that AP polypharmacy is common across geographical regions (although higher in Europe and Asia compared to North America) and time (47), despite the absence of strong evidence in its support apart from the recommendation for short-term treatment for TRS and/or cross-titration between antipsychotics (48). The safety and effectiveness of the combination of clozapine with other APs is a debated issue due to mixed findings: while some authors claimed that there is weak evidence to support clozapine polypharmacy (49), others suggest that some potential benefit (50–52) especially when combining it with aripiprazole in reducing the risk of hospitalization (52). However, our results suggested that AP dosages in the clozapine polytherapy group are not only above the recommended levels, which might pose concerns in terms of safety, but also do not bring any clinical benefit as no difference in symptoms between clozapine monotherapy and clozapine polytherapy was detected. Unfortunately, the study design did not enable investigation of the underlying reasons that influenced the prescription of clozapine monotherapy versus clozapine polytherapy. These may have included characteristics of the prescribers, patients and/or the facilities. It is thinkable that the severity of clinical symptoms could be higher in patients who were prescribed clozapine in combination with other antipsychotics; however, due to the cross-sectional nature of the study and the lack of access to prior/additional medical records, this type of investigation cannot be carried out. Finally, about psychotropic drugs other than APs, anxiolytics were prescribed in combination to clozapine in about 58% of the sample and antidepressants in 16%. The high prescription of anxiolytics reflects previous data from the region showing that benzodiazepines were prescribed in almost 82% of discharged patients with psychosis across nine SEE hospitals (53). Our result on the concomitant use of anxiolytics represents not only a violation of clinical guidelines but also a cause for concern given the proven association between increased use of benzodiazepines in patients with schizophrenia and a higher risk of mortality, whereas such an association was not found with antidepressants (54).

Overall, our findings suggest that there is non-adherence to clinical recommendations for clozapine prescription, with higher prescription rates yet in low dosages, which cannot be considered either therapeutic or evidence-based, and the prescription of clozapine in combination with one or more other AP medications. Although low adherence to clozapine prescriptions is a common international trend, more effort should be put to prevent off-label prescribing of clozapine. First, it has to be noted that in the five countries from this study, clinical guidelines for the treatment and management of schizophrenia exist only in Serbia and North Macedonia^1^, while there are not in Bosnia and Herzegovina, Kosovo by United Nations resolution, and Montenegro, which however use international guidelines as reference. Nevertheless, the existence of clinical guidelines for the treatment and management of schizophrenia does not translate into their implementation. Divac et al. showed that among Serbian psychiatrists, the decision about drug prescription was based on different factors such as drug safety (78%), efficacy (73%), clinical guidelines (65%), and reimbursement by health insurance (46%) (55). A more recent survey from Ignjatovic Ristic et al. (28) showed that concerns about potential side effects are the main obstacle to prescription (highest for possible agranulocytosis, 68%, and weight gain, 56%) (28). It was suggested that while in some western countries like the United Kingdom and the United States, clinical guidelines are backed by legislation, this is not the case in other regions (55).

Making guidelines mandatory would improve the chances of their wide implementation. However, such changes could be challenging without a unanimous effort involving relevant stakeholders and policy makers. Contextual factors, particularly in terms of deficiency of financial resources, in LMICs could be barriers to the implementation of guidelines, particularly for clozapine, which would require a high level of preparedness at the health system level (i.e., facilities, application of complex protocols for treatment, and constant monitoring). Examples of initiatives developed in the Netherlands (i.e., Dutch Clozapine Collaboration Group) and New Zealand aimed at increasing evidence-based knowledge on clozapine prescription (through regular audits and the inclusion of formal training on clozapine prescription for psychiatry residency) that resulted in increased clozapine’ prescription should be taken into account to implement clozapine prescription and adherence to guidelines (18). Similarly, patients have the right to make an informed decision about their treatment with clozapine, therefore, being educated about that would be an essential part of the process to guarantee engagement throughout the therapy (56).

We acknowledge the strengths and limitations of this study. It is the first multisite study including five LMICs in SEE looking specifically at clozapine prescription in a large sample of 401 patients with schizophrenia and schizoaffective disorders. It provides a naturalistic picture of the current prescription of clozapine in the region. However, as this is a cross-sectional study, it is not possible to state that the current prescription of clozapine is a maintenance therapy, as there is no longitudinal data collection or just a short-term dosage for cross-titration between antipsychotics. Furthermore, data on clozapine serum level was not available, thus, it could not be guaranteed that patients took what was prescribed. Indeed, a more accurate collection of data, possibly longitudinally, and a better understanding of psychiatrists’ attitudes toward clozapine prescription (potentially with a survey) should be done in order to gather a more precise picture of the pattern of clozapine use. Finally, while the five countries in this study include a good proportion of countries in the SEE region, the scenario might be different in other countries; thus, our results can be indicative rather than representative of the entire region.

## 5. Conclusion

In conclusion, despite rates of clozapine prescription across the five LMICs from SEE included in this study are higher compared to the international trend, low dosages indicate poor adherence to clinical guidelines. This could be due to several reasons, from the poor familiarity of clinicians with clozapine prescription to the lack of blood monitoring resources, and it might indicate that clozapine is prescribed mainly for its sedative effect rather than its antipsychotic. Future work should address this non-evidence-based practice. Indeed, it should be a priority for relevant stakeholders to improve clozapine use for patients’ benefit, enhance the quality of care, and possibly save costs on service care delivery if that is deemed necessary. The establishment of mandatory compliance to available (also international) clinical guidelines for the treatment of schizophrenia along with the provision of facilities and resources to allow monitoring of patients during clozapine treatment might represent the first step in promoting the use of clozapine for schizophrenia in SEE.

## Data availability statement

The raw data supporting the conclusions of this article will be made available by the authors, without undue reservation.

## Ethics statement

The studies involving human participants were reviewed and approved by the Bosnia and Herzegovina (Klinicki Centar Univerziteta u Sarajevu—Eticki Komitet 03-02-216, Eticki komitet JU Psihijatriska bolnica Kantona Sarajevo and JU Zavod za bolesti ovisnosti Kantona Sarajevo 02.8–408/19), Kosovo by United Nations resolution (Hospital and University Clinical Service of Kosovo—Ethics Committee 2019-85), Montenegro (Javna Zdravstvena Ustanova Klinicki Centar Crne Gore—Eticki komitet 03/01–29304/1, ZU Specijalna Bolnica za Psihijatriju Dobrota Kotor—Eticki komitet, Eticki Komitet JZU Dom Zdravlja Dr. Nika Labovic Berane 01-47), Republic of North Macedonia (Eticka Komisija za istrazuvanje na luge, Medicinski Fakultet pri UKIM vo Skopje 03-24219), and Serbia (Eticka komisija Medicinskog fakulteta u Beogradu 2650/XII-20 and Eticka komisija Specijalne bolnice Dr. Slavoljub Bakalovic Vrsac 01-36/1) and the United Kingdom (Queen Mary University of London QMREC2204a, 16 October 2018). The patients/participants provided their written informed consent to participate in this study.

## Author contributions

MR and NJ contributed to the study conception. MR contributed to data preparation, analysis, interpretation of results, and the first drafting of the manuscript. DI-R, DC, and NJ critically revised for important intellectual content. AA, SB, AD, LI, NM, and AN critically revised the final version of the manuscript. All authors read and approved the final version of the manuscript.

## Funding

Funding for this study was part of a larger study, which was provided by the European Union’s Horizon 2020 research and innovation program under grant agreement No 779334. The funding source had no involvement in study design, collection, analysis, interpretation of data, report writing, and decision to submit the manuscript for publication.

## Conflict of interest

DC declares to be a member of the non-profit organization the Dutch Clozapine Collaboration Group.

The remaining authors declare that the research was conducted in the absence of any commercial or financial relationships that could be construed as a potential conflict of interest.

## Publisher’s note

All claims expressed in this article are solely those of the authors and do not necessarily represent those of their affiliated organizations, or those of the publisher, the editors and the reviewers. Any product that may be evaluated in this article, or claim that may be made by its manufacturer, is not guaranteed or endorsed by the publisher.

## Footnotes

^1^ https://www.zdravlje.gov.rs/view_file.php?file_id=548&cache=sr

## References

[1] Huhn M Nikolakopoulou A Schneider-Thoma J Krause M Samara M Peter N. Comparative efficacy and tolerability of 32 oral antipsychotics for the acute treatment of adults with multi-episode schizophrenia: a systematic review and network meta-analysis. Lancet. (2019) 394:939–51. doi: 10.1016/S0140-6736(19)31135-3, PMID: 31303314 PMC6891890

[2] Souza JS Kayo M Tassell I Martins CB Elkis H. Efficacy of olanzapine in comparison with clozapine for treatment-resistant schizophrenia: evidence from a systematic review and meta-analyses. CNS Spectr. (2013) 18:82–9. doi: 10.1017/S1092852912000806, PMID: 23253621

[3] Bachmann CJ Aagaard L Bernardo M Brandt L Cartabia M Clavenna A. International trends in clozapine use: a study in 17 countries. Acta Psychiatr Scand. (2017) 136:37–51. doi: 10.1111/acps.12742, PMID: 28502099

[4] Krakowski M Tural U Czobor P. The importance of conduct disorder in the treatment of violence in schizophrenia: efficacy of clozapine compared with olanzapine and haloperidol. AJP. (2021) 178:266–74. doi: 10.1176/appi.ajp.2020.20010052, PMID: 33472389

[5] Tiihonen J Haukka J Taylor M Haddad PM Patel MX Korhonen P. A nationwide cohort study of oral and depot antipsychotics after first hospitalization for schizophrenia. Am J Psychiatry. (2011) 168:603–9. doi: 10.1176/appi.ajp.2011.10081224, PMID: 21362741

[6] Tiihonen J Lönnqvist J Wahlbeck K Klaukka T Niskanen L Tanskanen A. 11-year follow-up of mortality in patients with schizophrenia: a population-based cohort study (FIN11 study). Lancet. (2009) 374:620–7. doi: 10.1016/S0140-6736(09)60742-X, PMID: 19595447

[7] Tiihonen J Mittendorfer-Rutz E Majak M Mehtälä J Hoti F Jedenius E. Real-world effectiveness of antipsychotic treatments in a nationwide cohort of 29 823 patients with schizophrenia. JAMA Psychiat. (2017) 74:686–93. doi: 10.1001/jamapsychiatry.2017.1322, PMID: 28593216 PMC5710250

[8] Gören JL Rose AJ Smith EG Ney JP. The business case for expanded clozapine utilization. PS. (2016) 67:1197–205. doi: 10.1176/appi.ps.201500507, PMID: 27301766

[9] Oh PI Iskedjian M Addis A Lanctôt K Einarson TR. Pharmacoeconomic evaluation of clozapine in treatment-resistant schizophrenia: a cost-utility analysis. Can J Clin Pharmacol. (2001) 8:199–206., PMID: 11743592

[10] Remington G Addington D Honer W Ismail Z Raedler T Teehan M. Guidelines for the pharmacotherapy of schizophrenia in adults. Can J Psychiatr. (2017) 62:604–16. doi: 10.1177/0706743717720448, PMID: 28703015 PMC5593252

[11] Gee S Vergunst F Howes O Taylor D. Acta Psychiatr Scand. (2014) 130:16–24. doi: 10.1111/acps.12193 24004162

[12] Grover S Hazari N Kate N Chakraborty K Sharma A Singh D. Management of tardive syndromes with clozapine: a case series. Asian J Psychiatr. (2014) 8:111–114. doi: 10.1016/j.ajp.2013.12.016 24655641

[13] Daod E Krivoy A Shoval G Zubedat S Lally J Vadas L Psychiatrists’ attitude towards the use of clozapine in the treatment of refractory schizophrenia: A nationwide survey. Psychiatry Res. (2019) 275:155–161. doi: 10.1016/j.psychres.2019.03.029 30913436

[14] Tungaraza TE Farooq S. Clozapine prescribing in the UK: views and experience of consultant psychiatrists. Ther Adv Psychopharmacol. (2015) 5:88–96. doi: 10.1177/2045125314566808 26240748 PMC4521444

[15] Divac N Tosevski DL Babić D Djurić D Prostran M Samardzić R. Trends in consumption of psychiatric drugs in Serbia and Montenegro 2000-2004. Pharmacoepidemiol Drug Saf. (2006) 15:835–8. doi: 10.1002/pds.1316, PMID: 16958148

[16] Nielsen J Røge R Schjerning O Sørensen HJ Taylor D. Geographical and temporal variations in clozapine prescription for schizophrenia. Eur Neuropsychopharmacol. (2012) 22:818–24. doi: 10.1016/j.euroneuro.2012.03.003, PMID: 22503785

[17] Xu S-W Dong M Zhang Q Yang S-Y Chen L-Y Sim K. 2020. Clozapine prescription pattern in patients with schizophrenia in Asia: the REAP survey. Psychiatry Res. (2016) 287:112271. doi: 10.1016/j.psychres.2019.02.056, PMID: 30885383

[18] Bogers JPAM Schulte PFJ Van Dijk D Bakker B Cohen D. Clozapine underutilization in the treatment of schizophrenia: how can clozapine prescription rates be improved? J Clin Psychopharmacol. (2016) 36:109–11. doi: 10.1097/JCP.0000000000000478 26872120

[19] Cohen D. Prescribers fear as a major side-effect of clozapine. Acta Psychiatr Scand. (2014) 130:154–5. doi: 10.1111/acps.12294, PMID: 24841141

[20] Sanz-Fuentenebro FJ Uriarte JJU Bonet Dalmau P Molina Rodriguez V Bernardo Arroyo M. Pattern of use of clozapine in Spain. Variability and under-prescription. Rev Psiquiatr Salud Ment. (2019) 12:151–62. doi: 10.1016/j.rpsm.2018.02.005, PMID: 29631905

[21] Olfson M Gerhard T Crystal S Stroup TS. Clozapine for schizophrenia: state variation in evidence-based practice. PS. (2016) 67:152–2. doi: 10.1176/appi.ps.201500324, PMID: 26522679

[22] Patel MX Bishara D Jayakumar S Zalewska K Shiers D Crawford MJ. Quality of prescribing for schizophrenia: evidence from a national audit in England and Wales. Eur Neuropsychopharmacol. (2014) 24:499–509. doi: 10.1016/j.euroneuro.2014.01.014, PMID: 24491953

[23] Schou MB Drange OK Sæther SG. Differences between counties in the prescribing of clozapine. Tidsskr Nor Laegeforen. (2019) 139. doi: 10.4045/tidsskr.19.0151, PMID: 31556524

[24] Whiskey E Barnard A Oloyede E Dzahini O Taylor DM Shergill SS. An evaluation of the variation and underuse of clozapine in the United Kingdom. Acta Psychiatr Scand. (2021) 143:339–47. doi: 10.1111/acps.13280, PMID: 33501659

[25] Julaeha J Athiyah U Hermansyah A. The prescription patterns of second-generation antipsychotics in schizophrenia outpatient setting. J Basic Clin Physiol Pharmacol. (2019) 30:/j/jbcpp. doi: 10.1515/jbcpp-2019-0289, PMID: 31837257

[26] Mundt AP Aichberger MC Fakhriddinov S Fayzirahmanova M Grohmann R Heinz A. Prescription patterns of patients diagnosed with schizophrenia in mental hospitals in Tashkent/Uzbekistan and in four German cities. Pharmacoepidemiol Drug Saf. (2012) 21:145–51. doi: 10.1002/pds.2166, PMID: 21726013

[27] Szkultecka-Dębek M Miernik K Stelmachowski J Jakovljević M Jukić V Aadamsoo K. Treatment patterns of schizophrenia based on the data from seven Central and Eastern European Countries. Psychiatr Danub. (2016) 28:234–42., PMID: 27658832

[28] Ignjatovic Ristic D Cohen D Ristic I. Prescription attitudes and practices regarding clozapine among Serbian psychiatrists: results of a nationwide survey. Ther Adv Psychopharmacol. (2021) 11:204512532110202. doi: 10.1177/20451253211020235, PMID: 34104415 PMC8165825

[29] Ignjatović Ristić D Cohen D Obradović A Nikić-Đuričić K Drašković M Hinić D. The Glasgow antipsychotic side-effects scale for clozapine in inpatients and outpatients with schizophrenia or schizoaffective disorder. Nord J Psychiatry. (2018) 72:124–9. doi: 10.1080/08039488.2017.1400097 29125018

[30] Winkler P Krupchanka D Roberts T Kondratova L Machů V Höschl C. A blind spot on the global mental health map: a scoping review of 25 years’ development of mental health care for people with severe mental illnesses in central and Eastern Europe. Lancet Psychiatry. (2017) 4:634–42. doi: 10.1016/S2215-0366(17)30135-9, PMID: 28495549

[31] Jovanovic N Francis J Maric N Arenliu A Barjaktarov S Kulenovic A Implementing a psychosocial intervention DIALOG for patients with psychotic disorders in low and middle income countries in South Eastern Europe: protocol for a hybrid effectiveness-implementation cluster randomized clinical trial (IMPULSE). Global Psychiatry. (2019) 3:83–96. doi: 10.2478/gp-2019-0020

[32] Ventura J Lukoff D Neurchterlein Kh Liberman RP Green MF, Shaner A. Manual for expanded Brief Psychiatric Rating Scale. Int J Methods Psychiatr Res. (1993) 3:177–43.

[33] Kring AM Blanchard JJ Horan WP Reise SP. The clinical assessment interview for negative symptoms (CAINS): final development and validation. Am J Psychiatr. (2013) 170:165–72. doi: 10.1176/appi.ajp.2012.12010109 23377637 PMC3785242

[34] Priebe S Huxley P Knight S Evans S. Application and results of the Manchester Short Assessment of Quality of Life (MANSA). Int J Soc Psychiatry. (1999) 45:7–12. doi: 10.1177/002076409904500102 10443245

[35] Gardner DM Murphy AL O’Donnell H Centorrino F Baldessarini RJ. International consensus study of antipsychotic dosing. Am J Psychiatry. (2010) 167:686–93. doi: 10.1176/appi.ajp.2009.09060802, PMID: 20360319

[36] Leucht S Samara M Heres S Davis JM. Dose equivalents for antipsychotic drugs: the DDD method. Schizophr Bull. (2016) 42:S90–4. doi: 10.1093/schbul/sbv167, PMID: 27460622 PMC4960429

[37] Mortimer AM Singh P Shepherd CJ Puthiryackal J. Clozapine for treatment-resistant schizophrenia: National Institute of Clinical Excellence (NICE) guidance in the real world. Clin Schizophr Relat Psychoses. (2010) 4:49–55. doi: 10.3371/CSRP.4.1.4, PMID: 20643629

[38] Lehman AF Lieberman JA Dixon LB McGlashan TH Miller AL Perkins DO. Practice guideline for the treatment of patients with schizophrenia, second edition. Am J Psychiatry. (2004) 161:1–56. 15000267

[39] National Institute for Health and Excellence. Psychosis and Schizophrenia in Adults: Prevention and Management. London: National Institute for Health and Excellence. (2014). 32207892

[40] Olsson E Edman G Bertilsson L Hukic DS Lavebratt C Eriksson SV. Genetic and clinical factors affecting plasma clozapine concentration. Prim Care Companion CNS Disord. (2015) 17, 17:10.4088/PCC.14m01704. doi: 10.4088/PCC.14m01704, PMID: 26137357 PMC4468884

[41] Ruan C-J Zang Y-N Wang C-Y Cheng Y-H Sun C Spina E. Clozapine metabolism in East Asians and Caucasians: a pilot exploration of the prevalence of poor metabolizers and a systematic review. J Clin Psychopharmacol. (2019) 39:135–44. doi: 10.1097/JCP.0000000000001018, PMID: 30811372

[42] Suhas S Kumar V Damodharan D Sharma P Rao NP Varambally S. Do Indian patients with schizophrenia need half the recommended clozapine dose to achieve therapeutic serum level? An exploratory study. Schizophr Res. (2020) 222:195–201. doi: 10.1016/j.schres.2020.05.057, PMID: 32518001

[43] Meltzer HY. Clozapine treatment for Suicidality in Schizophrenia International suicide prevention trial (InterSePT). Arch Gen Psychiatry. (2003) 60:82–91. doi: 10.1001/archpsyc.60.1.82, PMID: 12511175

[44] Pardis P Remington G Panda R Lemez M Agid O. Clozapine and tardive dyskinesia in patients with schizophrenia: a systematic review. J Psychopharmacol. (2019) 33:1187–98. doi: 10.1177/0269881119862535, PMID: 31347436

[45] Victoroff J Coburn K Reeve A Sampson S Shillcutt S. Pharmacological management of persistent hostility and aggression in persons with schizophrenia spectrum disorders: a systematic review. JNP. (2014) 26:283–312. doi: 10.1176/appi.neuropsych.13110335, PMID: 26037853

[46] Volavka J Czobor P Nolan K Sheitman B Lindenmayer J-P Citrome L. Overt aggression and psychotic symptoms in patients with schizophrenia treated with clozapine, olanzapine, risperidone, or haloperidol. J Clin Psychopharmacol. (2004) 24:225–8. doi: 10.1097/01.jcp.0000117424.05703.29 15206671

[47] Gallego JA Bonetti J Zhang J Kane JM Correll CU. Prevalence and correlates of antipsychotic polypharmacy: a systematic review and meta-regression of global and regional trends from the 1970s to 2009. Schizophr Res. (2012) 138:18–28. doi: 10.1016/j.schres.2012.03.018, PMID: 22534420 PMC3382997

[48] Hasan A Falkai P Wobrock T Lieberman J Glenthoj B Gattaz WF. World Federation of Societies of Biological Psychiatry (WFSBP) guidelines for biological treatment of schizophrenia, Part 1: update 2012 on the acute treatment of schizophrenia and the management of treatment resistance. World J Biol Psychiatry. (2012) 13:318–78. doi: 10.3109/15622975.2012.696143, PMID: 22834451

[49] Barbui C Signoretti A Mule S Boso M Cipriani A. Does the addition of a second antipsychotic drug improve clozapine treatment? Schizophr Bull. (2009) 35:458–68. doi: 10.1093/schbul/sbn030, PMID: 18436527 PMC2659302

[50] Correll CU Rummel-Kluge C Corves C Kane JM Leucht S. Antipsychotic combinations vs monotherapy in schizophrenia: a meta-analysis of randomized controlled trials. Schizophr Bull. (2009) 35:443–57. doi: 10.1093/schbul/sbn018, PMID: 18417466 PMC2659301

[51] Taylor DM Smith L Gee SH Nielsen J. Augmentation of clozapine with a second antipsychotic - a meta-analysis: augmentation of clozapine with a second antipsychotic. Acta Psychiatr Scand. (2012) 125:15–24. doi: 10.1111/j.1600-0447.2011.01792.x, PMID: 22077319

[52] Tiihonen J Taipale H Mehtälä J Vattulainen P Correll CU Tanskanen A. Association of antipsychotic polypharmacy vs monotherapy with psychiatric rehospitalization among adults with schizophrenia. JAMA Psychiat. (2019) 76:499–507. doi: 10.1001/jamapsychiatry.2018.4320, PMID: 30785608 PMC6495354

[53] Maric NP Latas M Andric Petrovic S Soldatovic I Arsova S Crnkovic D. Prescribing practices in Southeastern Europe – focus on benzodiazepine prescription at discharge from nine university psychiatric hospitals. Psychiatry Res. (2017) 258:59–65. doi: 10.1016/j.psychres.2017.09.059, PMID: 28988045

[54] Tiihonen J Suokas JT Suvisaari JM Haukka J Korhonen P. Polypharmacy with antipsychotics, antidepressants, or benzodiazepines and mortality in schizophrenia. Arch Gen Psychiatry. (2012) 69:476–83. doi: 10.1001/archgenpsychiatry.2011.1532 22566579

[55] Divac N Marić NP Damjanović A Jovanović AA Jasović-Gasić M Prostran M. Use or underuse of therapeutic guidelines in psychiatry? Psychiatr Danub. (2009) 21:224–9., PMID: 19556953

[56] Verdoux H Quiles C. Educational needs and psychoeducation interventions in clozapine users: a narrative review. Acta Psychiatr Scand. (2020) 142:96–108. doi: 10.1111/acps.13172, PMID: 32266962

